# Plasma Rich in Growth Factors in the Treatment of Endodontic Periapical Lesions in Adult Patients: Case Reports

**DOI:** 10.3390/ijms22179458

**Published:** 2021-08-31

**Authors:** Katarzyna Machut, Agata Zoltowska, Elzbieta Pawlowska, Marcin Derwich

**Affiliations:** 1 Department of Endodontic Dentistry, Faculty of Medicine, Medical University of Gdansk, 80-210 Gdansk, Poland; katarzyna.machut@gumed.edu.pl (K.M.); agata.zoltowska@gumed.edu.pl (A.Z.); 2 Department of Orthodontics, Medical University of Lodz, 90-419 Lodz, Poland; elzbieta.pawlowska@umed.lodz.pl; 3 ORTODENT Specialist Orthodontic Private Practice in Grudziadz, 86-300 Grudziadz, Poland

**Keywords:** plasma rich in growth factors, platelet-rich fibrin, advanced platelet-rich fibrin, apical periodontitis, endodontics, endodontic treatment, bone regeneration

## Abstract

Platelet-rich fibrin (PRF) is an autologous blood concentrate obtained without anticoagulants by centrifugation of patients’ peripheral venous blood. PRF is considered to enhance the formation of new bone. The aim of this manuscript was to present two case reports of permanent teeth with closed apexes with periapical lesions, treated endodontically with the use of PRF. The root canals were mechanically cleaned and shaped with NiTi files and irrigated with 5.25% sodium hypochlorite (NaOCl), 40% citric acid (CA), and triple distillated water. Before the canal systems were obturated, A-PRF was used as a scaffold and was placed below the cementodentinal junction with hand pluggers. Cone beam computerized tomography (CBCT) was used to assess the resolutions of periapical radiolucencies. After 6 months, the measurements of both periapical lesions were significantly reduced. Although the performed root canal treatments (RCTs) can definitely be recognized as successful, it must be emphasized that mechanical shaping and cleaning of the root canals with special disinfecting solutions significantly affect the clinical efficacy of RCT. It seems impossible to state that PRF played a leading role in the healing process of the presented periapical lesions. Further studies must be performed to assess whether RCT of mature teeth with an additional PRF application is superior to RCT performed alone.

## 1. Introduction

Root canal treatment (RCT) is performed to eliminate pulpal infection, which may be the consequence of severe caries lesions or non-carious conditions, including traumas. The aim of RCT is to remove the inflamed or necrotic pulp, to chemo-mechanically debride the root canal system, and finally to hermetically fill the root canal system with biocompatible material [1].

Four conditions have been found that significantly improve the final outcome of primary RCT, including lack of the periapical radiolucency, homogenous filling of the root canal system, filling of the root canal system that extends to 2 mm within the radiographic apex, and well-sealing post-endodontic restoration [1]. Similar factors improve the final outcome of secondary RCT [2]. The major difficulty, which is related to secondary RCT, is the access to the apical infection. The final outcomes of primary and secondary RCT are similar when the access to the apical infection is restored [2].

RCT of teeth diagnosed with endodontic periapical lesions is associated with 49% lower odds of success compared with teeth without periapical lesions [2]. Therefore, it seems mandatory to search for new methods of treatment that may help the clinicians to achieve better outcomes in complicated clinical conditions.

Regenerative endodontic procedures (REPs) are based on biology and aim to replace impaired dental tissues, including dentin, pulp–dentin complex, and root structures. The major domains that affect regenerative endodontics are growth factors; stem cells; tissue engineering materials; and cell, tissue, and organ culture [3]. Several different techniques have been invented for regenerative endodontics; namely, injectable scaffold delivery, stem cell therapy, root canal revascularization, pulp implantation, scaffold implantation, three-dimensional cell printing, and gene delivery [3]. Nowadays, REPs are mostly performed in pediatric dentistry to treat immature teeth diagnosed with pulpal necrosis [4]. Although REPs for adult patients have also been discussed in the literature, little is known about their efficacy [5].

There are three major types of biological scaffolds used in REPs: blood clot revascularization (BCR), platelet-rich plasma (PRP), and platelet-rich fibrin (PRF) [6,7]. PRF is a second-generation platelet concentrate, which was developed by Choukroun in 2001 as a scaffold in maxillofacial surgery [8]. The fibrin in PRF has a structure of a three-dimensional network, which is a flexible, elastic, and makes a very strong core, in which platelets and leukocytes are suspended [8].

According to the available literature, PRF seems to be very effective in regenerative dentistry [9]. It is considered to enhance the formation of a new bone [10]. Therefore, it may be speculated that the application of PRF in the apical region, before final obturation of the root canal system, may accelerate the regeneration of periapical tissue. There is no evidence of data in the worldwide literature concerning the use of PRF in the endodontic treatment of mature permanent teeth with diagnosed chronic periapical periodontitis.

The aim of this manuscript was to present two case reports of permanent teeth with closed apexes diagnosed with periapical lesions, treated endodontically with the use of PRF.

## 2. Case Report—Patient A: Pulp Necrosis with Symptomatic Apical Periodontitis

A 45-year old female patient came to the Department of Endodontic Dentistry Medical University of Gdansk for a severe pain of tooth 23 (upper left canine, according to the Federation Dentaire Internationale (FDI) dental numbering system). Extraoral and intraoral examinations were carried out. The gingiva above tooth 23 was found to be swollen, reddish, and painful to palpation. Tooth 23 showed Grade 3 mobility (Miller’s index of mobility). Examination of the periodontal pocket revealed the presence of exudation, which mostly consisted of pus. The pocket depth (PD) was PD max = 9 mm. Diagnostic examination of a pulp viability was performed with faradic current. Tooth 23 did not respond to electric stimuli, which is typical for nonvital teeth. The periapical tissue condition was additionally tested by the reaction to vertical and horizontal percussion. Both of the reactions were positive.

**Figure 1:**
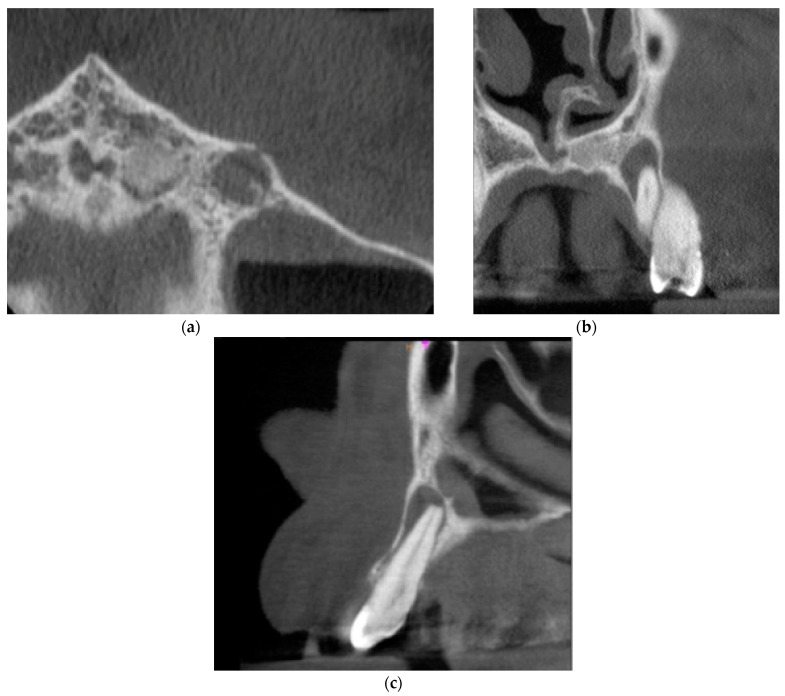
Preoperative cone beam computerized tomography (CBCT) images presenting the periapical lesion around the root of tooth no. 23 (date of CBCT examination: 22 May 2020): (**a**) axial view; (**b**) coronal view; and (**c**) sagittal view.

Cone-beam computed tomography (CBCT) was performed. The periapical lesion was measured with the use of CS 3D Imaging v3.5.18 Software (Carestream Health Inc., Trophy, Croissy-Beaubourg, France). The measured dimensions of the lesion were 9.0 × 7.2 × 9.9 mm. The CBCT images of the periapical lesion are presented in Figure 1.

The patient was diagnosed with pulp necrosis with symptomatic apical periodontitis of tooth 23 and qualified for an endodontic treatment. Patient informed consent to per-form root canal treatment was obtained. The endodontic treatment was performed under local anesthesia. The rubber dam was placed before the onset of the endodontic treatment. The root canal was prepared chemo-mechanically with the modified crown-down technique using Nickel Titanium (NiTi) 0.04 rotary instruments (K3, Kerr, Glendora, CA, USA) on a working length. The working length (WL) was 27 mm and was confirmed both with the indication of the endometer (Raypex 5, VDW, Munich, Germany) and radiologically. Apical gauging was determined with 0.02 hand K-file NiTi ISO 30, so the diameter of apical foramen measured 0.30 mm. The root canal irrigation protocol along with ultrasonic activation (PUI) included the following: 5.25% sodium hypochlorite (NaOCl), 40% citric acid (CA), and triple distilled water. Between the following instruments, the root canal was irrigated with 2 mL of NaOCl with PUI. After the final shape was completed, the root canal was rinsed with 10 mL of NaOCl and 5 mL of CA. The final rinse was carried out with 2 mL of NaOCl and 2 mL of triple distilled water. Every time, the irrigation needle was placed at a depth of 2 mm from the apex.

**Figure 2:**
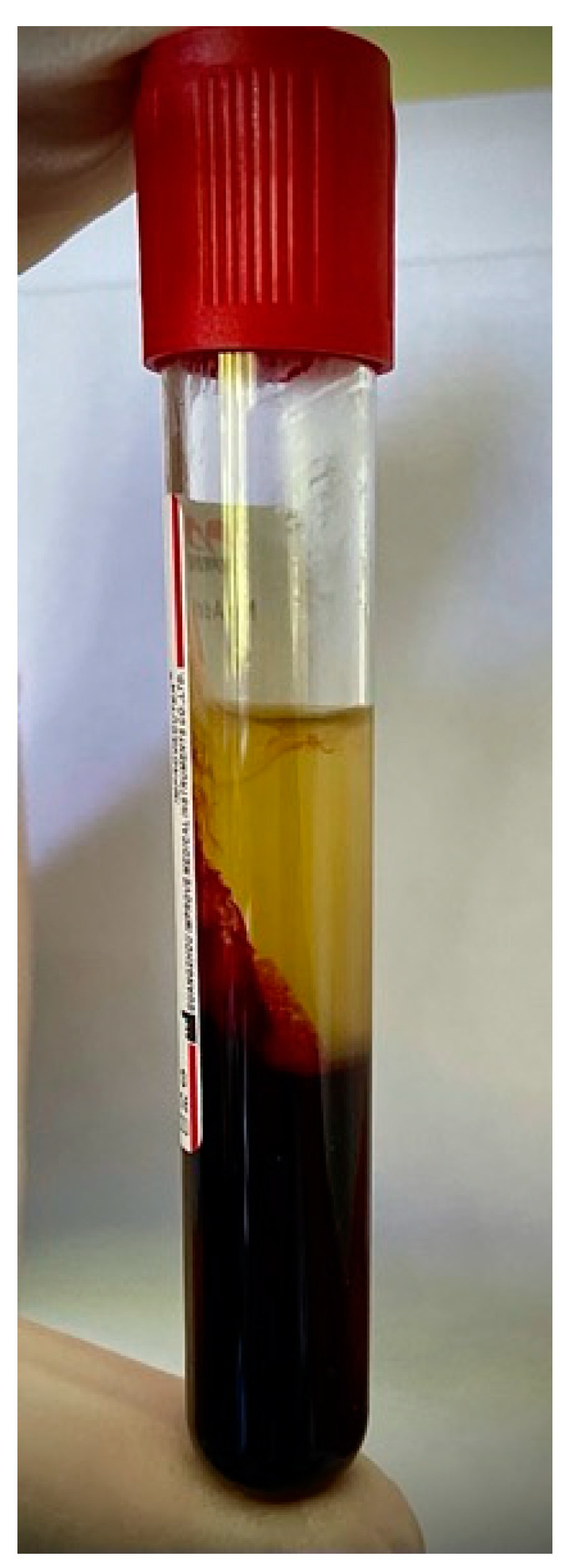
The probe with the obtained advanced platelet-rich fibrin (A-PRF) after centrifugation.

The patient’s blood was drawn from a median cubital vein and collected in glass tubes (each 10 mL). Next, the patient’s blood was centrifugated at 1200 rpm for 8 min in the Neuation iFuge D06 Premium Edition (Neuation Technologies Pvt., Gandhinagar, Gujarat, India) centrifuge to obtain advanced platelet-rich fibrin (A-PRF). Figure 2 presents the probe with the obtained A-PRF after centrifugation.

Using sterile tweezers, the fibrin clot was squeezed between two gauze pieces to create an autologous fibrin membrane. After the root canal was dried with paper cones, the freshly prepared A-PRF membrane was placed into the apex and then pushed below the level of the cementodentinal junction using Machtou hand pluggers—size 1/2 NiTi (red) and 3/4 (grey).

**Figure 3:**
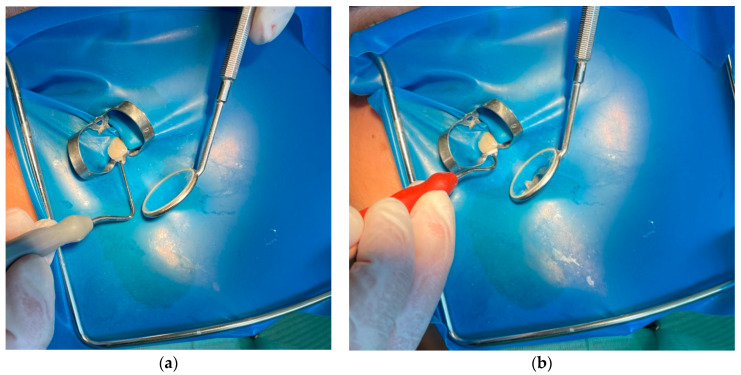
Application of A-PRF with a Machtou hand plugger: (**a**) initial A-PRF application with a grey Machtou hand plugger; (**b**) application of A-PRF with a red Machtou hand plugger below the cementodentinal junction.

Figure 3 presents the application of the A-PRF into the root apex of tooth no. 23. 

Finally, the root canal was filled by a thermoplastic method (BeeFill 2in1 Obturation Kit, VDW GmBH, Munchen, Germany) with calibrated gutta-percha cone MAC ISO 30.04 and AH-plus sealer (Dentsply DeTrey GmbH, Philadelphia, PA, USA), using the combination of a Downpack heat source with a Backfill extruder. The methodology of the endodontic treatment was based on the guidelines of the American Association of Endodontists and the European Society of Endodontology [11,12]. Follow-up appointments took place after 1 week, 3 months, and 6 months.

The first follow-up appointment was conducted 1 week after the end of the treatment. The patient reported that the pain after the treatment lasted for 24 h. There were no symptoms of an acute inflammation in the intraoral examination. The gingiva was smooth, pink, and humid, with no pain on palpation. The mobility of the tooth was reduced to Grade 2 Miller mobility index. PD max = 9 mm. The reaction to vertical and horizontal percussion was still positive.

The second check-up was carried out 3 months after the end of the treatment. The patient reported that there had been no incidents of pain during that period. No features of inflammation were noticed during the examination. The gingiva was smooth, pink, and humid, with no pain on palpation. The mobility of the tooth was within the physiological limits (Grade 1 Miller mobility index). PD max = 7 mm. The reaction to vertical percussion was positive, but the reaction to horizontal percussion was negative.

**Figure 4:**
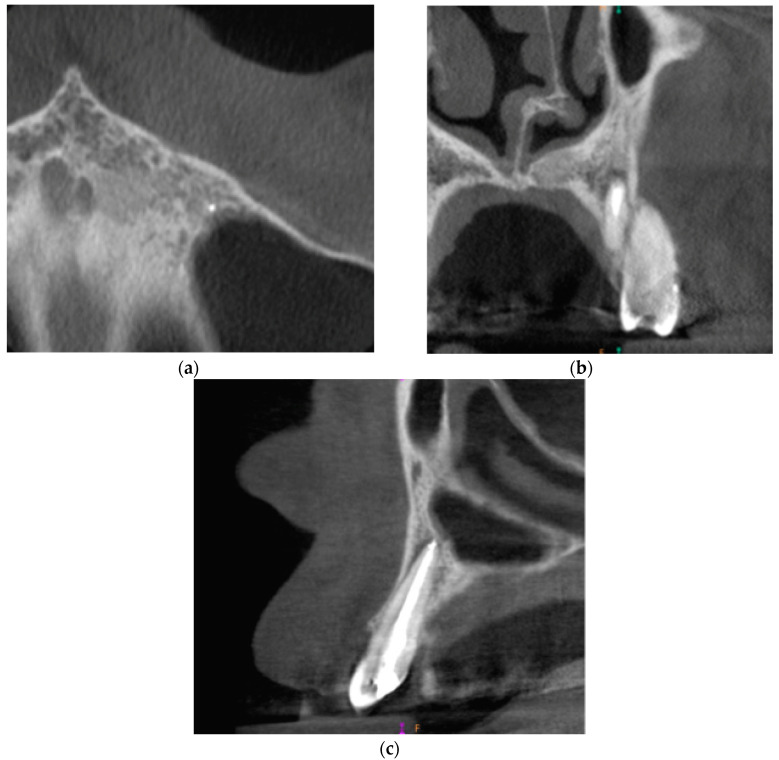
CBCT images presenting the healing process of the periapical lesion around the root of tooth no. 23 (date of CBCT examination: 8 December 2020): (**a**) axial view; (**b**) coronal view; and (**c**) sagittal view.

The third and final appointment was scheduled 6 months after the end of root canal treatment. Intraoral examination revealed healing progression. The gingiva was smooth, pink, and humid, with no pain on palpation. The mobility of the tooth was within the physiological limits (Grade 1 Miller mobility index). The reaction of tooth 23 to vertical and horizontal percussion was negative. PD max = 4 mm. The CBCT images presented healing of the periapical lesion. There was a small area of radiolucency around tooth no. 23 with the dimensions of 2.6 × 1.0 × 0.6 mm. Figure 4 presents the CBCT images of the periapical lesion healing process 6 months after the end of endodontic treatment.

## 3. Case Report—Patient B: Pulp Necrosis with Asymptomatic Apical Periodontitis

A 42-year old male patient came to the Department of Endodontic Dentistry Medical University of Gdansk because of caries in tooth 23 (upper left canine, according to the FDI dental numbering system). The patient was generally healthy. Medical history and extraoral examination were not significant. No soft-tissue abnormality was found in the intraoral examination. Tooth 23 presented Grade 2 Miller mobility index. There was no exudation from the periodontal pocket. The pocket depth was measured; PD max = 3 mm. Tooth no. 23 did not respond to the electric stimuli, which confirmed the tooth was not vital. The reaction to vertical percussion was positive, whereas the reaction to horizontal percussion was negative.

**Figure 5:**
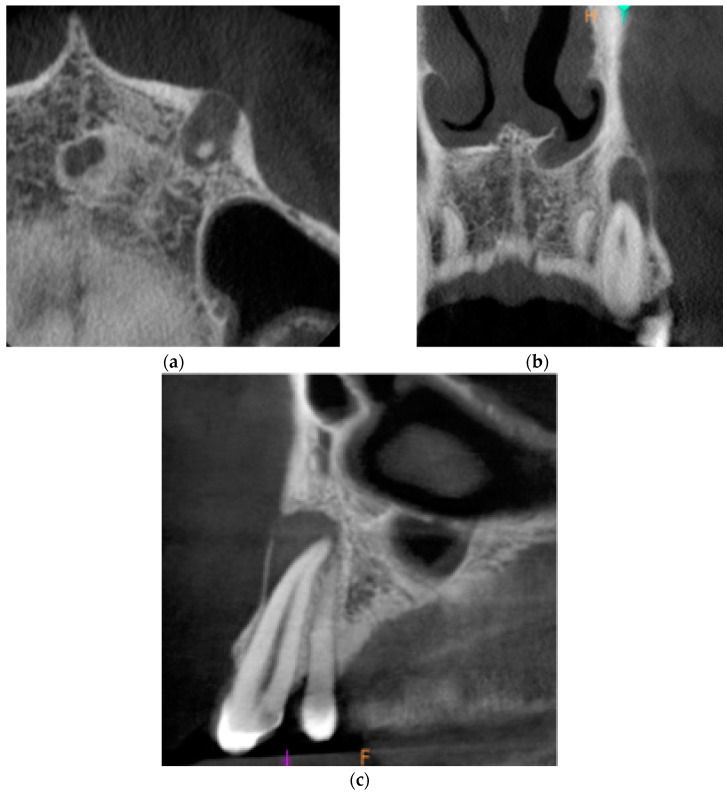
Preoperative CBCT images presenting the periapical lesion around the root of tooth no. 23 (date of CBCT examination: 19 January 2021): (**a**) axial view; (**b**) coronal view; and (**c**) sagittal view.

The CBCT of tooth 23 was taken. The periapical lesion was also measured with the use of CS 3D Imaging v3.5.18 Software (Carestream Health Inc., Trophy, Croissy-Beaubourg, France). The measured dimensions of the lesion were 12.7 × 8.7 × 6.4 mm. The CBCT images of the periapical lesion are presented in Figure 5.

The patient was diagnosed with pulp necrosis with asymptomatic apical periodontitis of tooth 23. The endodontic treatment was performed with the modified crown-down technique, using NiTi 0.04 rotary instruments (K3, Kerr, Glendora, California, USA) on a working length. The working length (WL) in this case was 29 mm and apical gauging was measured by hand 0.02 K-file ISO 30 and 35, so the diameter of the apical foramen was larger than 0.3 mm, but smaller than 0.35 mm. A-PRF was applied in the same manner as in Patient A. Then, the root canal was filled by a thermoplastic method (BeeFill 2in1 Obturation Kit, VDW GmBH, Munchen, Germany) with calibrated gutta-percha cone MAC ISO 35.04 and AH-plus sealer (Dentsply DeTrey GmbH, Philadelphia, PA, USA). There were also three follow-up appointments, after 1 week, 3 months, and 5 months.

One-week follow-up: no pain after the treatment was reported. No abnormalities of the soft tissue were noticed. The mobility of the tooth was within the physiological limits (Grade 1 Miller mobility index). The reaction to vertical percussion was positive.

Three months after the treatment: no incidents of pain were observed by the patient. During intraoral examination, the gingiva was smooth, pink, humid, and painless. The mobility of the tooth was within the physiological limits (Grade 1 Miller mobility index). The reaction to vertical percussion was negative.

**Figure 6:**
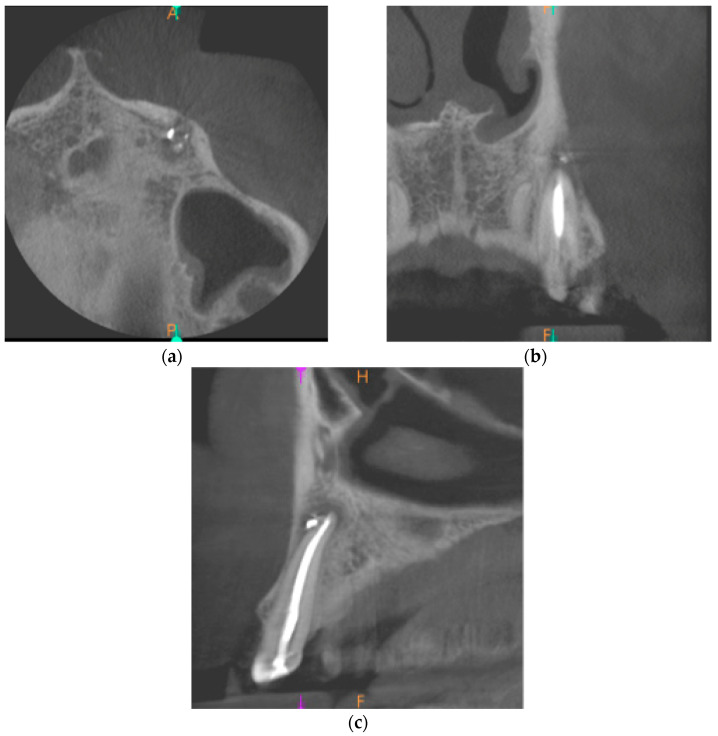
CBCT images presenting the healing process of the periapical lesion around the root of tooth no. 23 (date of CBCT examination: 29 June 2021): (**a**) axial view; (**b**) coronal view; and (**c**) sagittal view.

Finally, 5 months after the end of the treatment, there was no evidence of any pathological symptoms. The CBCT images revealed the process of healing of the periapical lesion. There was a small area of radiolucency around tooth no. 23 with the reduced dimensions of 4.6 × 4.6 × 2.1 mm. Figure 6 presents the CBCT images of the healing process of the periapical lesion 5 months after the end of endodontic treatment.

## 4. Discussion

Within this article, two clinical cases diagnosed with periapical lesions are presented. Both of the cases were treated with conventional RCT (with the principles of the American Association of Endodontists and the European Society of Endodontology) with an additional A-PRF application by the apical foramen to the periapical area. Six months after the end of the performed endodontic treatment, healing of the periapical lesions was noticed. 

Despite the fact that the performed endodontic treatments can definitely be recognized as successful, it must be emphasized that mechanical shaping and cleaning of the root canals with special disinfecting solutions significantly affect the clinical efficacy of the endodontic treatment. These procedures lead to elimination of the pathogens from the root canals [13,14,15]. Sabeti et al. [16] emphasized that healing of the periapical tissues strongly depends on the proper decontamination of root canal systems, host immune response, and good coronal seal, which may be obtained with the properly prepared crown restoration.

Moreover, it is known that apical papilla stem cells (SCAPs) are able to survive at apical periodontitis and may even further develop after an endodontic infection [17,18]. SCAPs promote the growth of new tissues [18,19]. They present osteogenic potential and increase angiogenesis [18]. On the basis of the presented cases, it seems impossible to state if and how PRF affected SCAPs’ activity, as well as what exactly the role of SCAPs was in the healing process of the periapical lesions.

Therefore, although the presented cases look very promising, it seems impossible to state that PRF played the leading role in the healing process of the presented periapical lesions. Further studies, especially randomized, double-blind controlled trials, must be performed to assess if the endodontic treatment of permanent, mature teeth with periapical lesions with an additional PRF application is superior to the endodontic treatment performed alone.

According to the guidelines of the European Society of Endodontology, periapical lesions should be observed for a minimum of four years. If the area of radiolucency remained the same size or the size changes are indiscernible, the endodontic treatment is recognized as a failure and an additional treatment is required, including endodontic surgery, or even tooth extraction [11]. Zhang et al. [20] radiographically analyzed the size of the periapical lesions for two years after RCT had been completed. The authors noticed that 92% of the examined teeth presented reduced areas of radiolucency 1 year after RCT. Two years after RCT, in 63% of cases, further reduction of the periapical lesions was observed; in 33% of cases, the periapical lesions remained unchanged; and in 3% of the analyzed cases, the volume of periapical lesions increased. Zhang et al. [20] concluded that healing of periapical lesions is a dynamic, long-lasting process.

Autologous platelet concentrates have been widely used in regenerative endodontics for the treatment of immature teeth. It has been proven that platelet concentrates are able to stimulate apical closure [21]. Although the impact of PRF on healing of the periapical lesions in immature necrotic teeth has been discussed by many researchers, the results are not unequivocal [22,23]. PRF may be considered as an ideal bioscaffold to increase proliferation and differentiation of cells that take part in the process of tissue repair [24]. Moreover, it has been found that reduction of the relative centrifugal force improves the regenerative potential of the PRF-based matrices [25]. A-PRF and A-PRF+ are the modifications of PRF that are obtained with the principles of the low speed centrifugation concept (LSCC) [26].

So far, there have not been any manuscripts published analyzing the clinical effects of conventional RCT with supplementary PRF application in the treatment of periapical lesions in mature teeth. Only few case reports have been presented of permanent, mature teeth with periapical lesions treated with RCT combined with surgical procedures, including curettage of the defect and sometimes apical resection [27,28,29,30,31,32,33,34,35,36,37,38]. The defects were filled with either platelet concentrate alone or platelet concentrate mixed with bone substitutes [27,28,29,30,31,32,33,34,35,36,37,38]. Despite the fact that all of the published cases were successful, they do not support the exact role of PRF in the process of healing. Parikh et al. [38] presented an interesting case of a patient with exacerbated chronic periodontitis in relation to nonvital teeth 11 and 21, treated with RCT and curettage of the defect. PRP gel was placed only at the site of the larger defect (left side). The authors observed that the side with PRP healed better compared with the other side.

## 5. Conclusions

RCT combined with an additional application of A-PRF (by the apical foramen to the periapical area), performed in permanent, mature teeth diagnosed with periapical lesions, led to a significant decrease in the periapical lesions’ size within six months. However, these observations do not explain the exact role of the A-PRF in the process of healing. 

## Data Availability

The data presented in this study are available on request from the corresponding author.
